# Consenting rather than choosing. A qualitative study on overseas patients' decision to undergo hematopoietic stem cell transplantation

**DOI:** 10.1002/cam4.6934

**Published:** 2024-01-09

**Authors:** Aline Sarradon‐Eck, Loreley Franchina, Yolande Arnault, Anne‐Gaëlle Le Corroller, Patricia Zunic, Patricia Marino

**Affiliations:** ^1^ INSERM, IRD, SESSTIM, ISSPAM Aix Marseille Université Marseille France; ^2^ CanBios UMR1252 Institut Paoli‐Calmettes Marseille France; ^3^ Département de Psychologie Clinique Institut Paoli‐Calmettes Marseille France; ^4^ Service d'hématologie et d'oncologie médicale CHU La Réunion Saint Pierre France

**Keywords:** allogeneic hematopoietic stem cell transplantation, bone marrow transplantation, France, informed consent, La Réunion, patients' decision making, qualitative study

## Abstract

**Objective:**

Reasons for patients' acceptance of the allogeneic hematopoietic stem cell transplantation (allo‐HSCT) proposed and how their decision may be affected by the long distances involved have not been sufficiently investigated so far. We therefore conducted a qualitative study to identify the factors involved in overseas patients' decision to accept allo‐HSCT.

**Methods:**

In‐depth semi‐directive interviews were conducted with overseas allo‐grafted patients (*n* = 22), as well as one non‐consenting patient and their caregivers (*n* = 24). Interviews were analyzed taking an inductive thematic approach.

**Results:**

Respondents stated that their decision to undergo the transplantation was constrained by their feeling of being in a therapeutic impasse, the need for a survival strategy, the need to survive for their family's sake, family and doctors' pressures, and the feeling of being managed. The following factors favoring patients' acceptance were the medical information received, their faith, having a family donor, peer testimonies, and positive representations of the transplantation. Factors against patients' acceptance were geographical distance from home to the transplant center, apprehension of protective isolation, fear of dying, and representations of the graft.

**Conclusions:**

These factors, such as patient's personal values and representations, need to be weighed up in order to adapt the information exchanged accordingly. Efforts are required to relieve patients' social isolation and improve the means of providing family support.

## 1 INTRODUCTION

In this study, we investigated why patients with hematological cancer consent to undergo allogeneic hematopoietic stem cell transplantation (allo‐HSCT). This standard treatment is a curative treatment for several forms of hematological cancer with an unfavorable prognosis and several other hematological disorders. However, allo‐HSCT, which has potentially serious long‐term life‐threatening side effects (such as acute and chronic graft‐versus‐host disease) and involves a high risk of treatment‐related death, requires patients to give their informed prior consent. Informed consent refers to both the patient's right to self‐determination and physicians' obligation to inform their patients.^1^ However, in the context of a life‐threatening disease for which there exist no curative alternatives, some authors have argued that patients' consent to a life‐saving intervention such as allo‐HSCT is either constrained^1^ or at least resigned.^2^ Studies on patients' decision to undergo allo‐HSCT have mainly focused on the ethical aspects of the informed consent process. Several authors have studied patients' satisfaction with this process^3^, ^4^ and how to ensure that patients' decision will be sufficiently “well informed”.^5^, ^6^, ^7^, ^8^ Various guidelines to obtaining informed consent have been published.^2^, ^9^

Several authors have examined patients' understanding of the therapeutic process and the risks involved. It has emerged that patients may tend to overestimate the benefits of the transplantation,^7^, ^10^, ^11^ and the two main factors favoring acceptance seem to be their perception that allo‐HSCT is the only available means of surviving longer and their trust in their physicians.^1^, ^3^, ^5^ In addition, Forsyth et al.^5^ have stressed the role of social factors, such as patient/doctor relationships, the pressures exerted by patients' family and social network and their willingness to recover their previous social roles, especially their family roles.

Another factor involved in the decision seems to be a long distance from home to the specialized hospital, as observed in Canada^12^ and Australia.^13^ Geographical distance has also been mentioned by hematologists in France as a factor liable to influence patients' and physicians' decision‐making in the case of patients inhabiting French overseas regions.^14^, ^15^

However, the fact that some patients accept allo‐HSCT while others do not, and how patients' decision may be affected by the long distances involved are two issues which have not yet been thoroughly investigated. To our knowledge, no data are available on the numbers eligible for allo‐HSCT who refused the transplantation. In a situation in which patients and clinicians may have different values and preferences, it is important to increase the knowledge about what guides decision‐making from the patients' point of view in order to help physicians inform their patients more clearly about the treatment proposed and how the allograft may affect their lives, especially in the case of patients inhabiting remote areas. Furthermore, the fact that allo‐HSCT is the only curative treatment available for a number of hematological pathologies makes the question of therapeutic acceptance particularly relevant.

We therefore conducted a qualitative study, using an anthropological “lived experience” approach^16^ to identify what determines patients' decision to accept allo‐HSCT. We interviewed patients inhabiting the island of La Reunion (a French overseas Department located in the Indian Ocean, more than 9000 km from Paris) who were candidates for allo‐HSCT in mainland France. At the time of our study, the only transplant centers available were located in mainland France. In a previous article,^17^ we described how overseas therapeutic mobility affects patients' and caregivers' experience of the transplantation. Here we focused on patients' decision to undergo allo‐HSCT and the main psychosocial, cultural, and geographical factors involved in this process.

## 2 METHODS

This qualitative study was undertaken as scoping work for a broader research programme, using qualitative and quantitative methods to address the psychosocial consequences of geographical distance on patients and their family caregivers, the reasons for patients' decision to undergo an allo‐HSCT overseas or not, and the economic consequences of the current conditions under which patients are transferred to mainland France.

This qualitative study was approved by the French National Institute for Health and Medical Research Institutional Review Board (IRB00003888, No. 19‐631). Patients and family caregivers were invited to participate after receiving both oral and written information about the study. All information about the participants was anonymized to ensure strict confidentiality.

### 2.1 Participants

Were conducted in‐depth semi‐directive interviews between February 2020 and January 2021 with the following participants: 22 allo‐HSCT recipients (coded P), one non‐consenting patient (coded PR1), and 24 family caregivers (coded C, and CR1 in the case of the non‐consenting patient's carer) (Table 1). For data triangulation purposes, nine health professionals working at La Reunion hospital's partner transplant center were also interviewed.

**TABLE 1:** Patients' and carers' characteristics.

Patients (*n* = 23)	
Patients who had undergone allo‐HSCT	22
Not consenting patients	1
Age (years) at the time of the interview (*n* = 23)	
20–29	3
30–39	6
40–49	3
50–59	5
60–69	4
≧70	2
Gender (*n* = 23)	
Male	13
Female	10
Diagnosis (*n* = 23)	
Acute myeloid leukemia	13
Lymphoma	3
Acute lymphoblastic leukemia	4
Myelodysplasic syndrom	2
Aplastic anemia	1
Duration of transfer (months)	
Median	4.73 [2.5–11]^a^
Times after (first) HSCT at the time of the interview (years) (*n* = 22)	
<1	8
1–2	8
2–3	3
3–4	1
4–5	2
≥5	0
Type of donor (*n* = 22)	
Related	15
Unrelated	7
Origins (*n* = 23)	
Reunionese	18
Mainland France	3
Mayotte	2
Number of children (*n* = 23)	
0	3 respondents
1	7 respondents
2	7 respondents
≥3	6 respondents
Marital status (*n* = 23)	
Single	5
Partnered	16
Divorced	1
Widowed	1
Occupation at the time of diagnosis (*n* = 23)	
Employed	9
Self‐employed	3
Students	2
Not employed	5
Retired	4
Caregivers (*n* = 24)	
Caregivers of allo‐grafted patients	23
Caregivers of non‐consent patients	1
Age (years) at the time of the interview	
20–29	2
30–39	4
40–49	6
50–59	8
60–69	3
≥70	1
Gender	
Male	6
Female	18
Relationship with the Patients	
Mother	6
Father	1
Wife	4
Husband	1
Daughter	2
Siblings	4
Siblings in‐law	2
Friends/Neighbors	4
Occupation at the time of the interview	
Employed	9
Self‐employed	3
Not employed	10
Retired	2

^a^ The last patients transferred had a shorter stay in mainland France.

### 2.2 Study procedure

Patients were recruited in collaboration with the Reunion University Hospital (CHU‐S). They were sampled consecutively without any selection criteria other than being over 18 years of age and having been a transplant candidate. Hematologists at the CHU‐S gave us a list of 14 patients who had refused the graft from 2014 to 2020. Seven of them had died meanwhile and two were lost from follow‐up. Four of the five non‐consenting patients contacted declined to participate in the study. Patients provided themselves carer contact information.

The interviews were conducted in person at the participants' homes or in a hospital room by an anthropologist (LF) living in La Reunion. However, because of the COVID‐19 pandemic, some interviews were conducted by telephone and video with some of the participants (six patients, eight caregivers, and nine health professionals).

Interviews with patients and carers covered a wide range of topics relating to their experience of the transplant procedure, the period of isolation involved, the participants' understanding of the disease and the treatment, and the factors involved in the patients' decision. The interview guides used are presented in the Data S1.

The interviews were audio‐recorded and faithfully transcribed verbatim. All personally identifiable details were removed from the transcripts.

### 2.3 Data analysis

This qualitative study, with an anthropological approach, does not aim to product quantitative data, but a “thick description” of patients' point of view, values, and preferences This approach does not allow generalization but does allow some degree of transferability.^18^ We conducted a qualitative analysis of each interview using a Grounded Theory analysis.^19^ The first 10 transcripts were coded by two coders (the first and second authors, who are medical anthropologists) working independently and using an iterative inductive process in the MAXQDA environment. We compared the coding of each transcript. The discrepancies between the two were discussed until the final coding scheme was adopted. The few differences in the interpretation of data were examined in relation to the existing literature in order to spot any inexactness or misinterpretations. The second author then conducted the coding of all the interviews.

During the initial coding process, sections of interviews were sorted into various categories until the categories accounted for all the variations in the data. At the second step (focused coding), we refined selective coding and relationships between the categories. We identified core categories and arranged them into broad emergent thematic categories and sub‐themes. The analyses presented here were based on a particular subset in the coding framework, in which narratives relating to patients' decision‐making were identified. This coding framework and excerpts from some of the participants' interviews which illustrate each sub‐theme are presented in Data S2.

The following strategies were applied in order to ensure the rigor of the analysis^20^: saturation, data triangulation between patients' and caregivers' data, and reflexivity. We conducted simultaneously the data collection and the analysis until no new items appeared (i.e., until theoretical saturation was reached).^19^ We constantly checked, compared and contrasted the data codes, categories and themes, and discussed them at interdisciplinary team meetings (including two anthropologists, a psychologist, a hematologist, and two health economists).

## 3 RESULTS

The following main themes emerged from the analysis. They focused on obviousness of choice and factors influencing patients' decision‐making.

### 3.1 A constrained choice

From the doctors' point of view, consenting to an allograft corresponded to validating the treatment proposed, whereas most of the patients expressed their views in terms of choice. “We had no choice” was a statement frequently made during both patients' and caregivers' interviews. The idea that there really was no choice can be interpreted and explained in several ways as follows.

#### 3.1.1 The feeling of being in a therapeutic impasse

The transplant was “the only solution,” meaning that it was the only realistic treatment option available, which the patients had approved during their discussions with their doctors. Some of them stated that the only other options were either “doing nothing at all” or undergoing a treatment which would have little effect on their disease.

#### 3.1.2 A survival strategy

The respondents felt that the choice to have the transplantation was ineluctable despite the uncertainty surrounding the treatment. The choice between life and death therefore replaced the common dilemma based on the benefit/risk ratio: “It's a question of opting for life” (P4). In this situation, most of the respondents overlooked or minimized the weight of possible complications and the risk of transplantation failure:

When you know you are going to die otherwise, what risk are you taking? No risk at all. I always knew there would be a few little problems (complications). Not such big ones, though. But I'm still here, I'm still alive. (P19)

#### 3.1.3 Surviving for their family's sake

In patients' strategy for survival, both affective factors and factors relating to the individual's social status were at work, especially their parental role, with which 20 of the 23 patients in our sample were concerned. Those with young children said they hoped to survive in order to resume their parental role:

I want to go on living. My fight is for living. Living not just for myself, but for him (her son) because I must help him to live. Because I want to pass on everything I know to him. (P18)

#### 3.1.4 Family pressures

Patients' families played a decisive role when the patients themselves were hesitating or refusing the transplantation. In addition to the support they provided throughout the whole process, some of the patients' parents and children weighed heavily on their decision. One patient aged 22 declared, however, that he had reached his decision quite independently:

My father didn't want me to have it (allo‐HSCT) but my mother wanted me to have it. It's a decision I took all on my own, a well thought‐out decision. (P22)

#### 3.1.5 Doctors' pressures

The respondents mostly stated that their doctors were quite insistent in order to convince patients and their families that allo‐HSCT was worth being undergone. The doctors interviewed were well aware of their powers of persuasion.

#### 3.1.6 Being managed

The way patients are managed because of the urgency of the situation and the uncertainty about the evolution of the disease and the efficacy of the treatment gave most of the patients the feeling (which tended to be reassuring) that they were caught up in a process with no other choice but to comply with the doctors' orders:

To my mind, it was part of the whole procedure, one just had to go along with it. At one point, I wondered whether I should really do it or not. He (the physician) said to me ‘Of course you must do it, that's how it is and there's no other way about it’(…). There was no shilly‐shallying. (P12)

### 3.2 Factors involved in patients' decision to accept allo‐HSCT

#### 3.2.1 The semantic register of the decision‐making

Paradoxically, although the patients and caregivers said they consented to the transplantation only with some resignation, the semantic content of their narratives often suggested that decision‐making was an active, rational process: “deciding”, “forging ahead”, “wanting”, “putting all the chances on one's side”, “playing an active part” in their treatment rather than just “undergoing it”. The semantic register was also that of confidence in the medical teams, which sometimes took the form of a “moral contract” or a “healing contract”. Lastly, several respondents felt they had been able to choose between two therapeutic options, which helped them express their preferences in an informed decision‐making process.

Our analysis yielded various factors positively or negatively affecting patients' decision to undergo an allo‐HSCT.

#### 3.2.2 Factors favoring patients' acceptance

##### The medical information received

Most of the respondents declared that they had received enough information from their doctors, felt they had made the right choice, and were especially grateful to them:

To all the questions I asked they gave me the answers. And even before asking these questions, I already had the answers, and that was…really important. (…) Thanks to these discussions, I never had the feeling that I was going to die (…). The idea that it might not work just never entered my mind. I am sure it was thanks to the doctors. They managed to advise and thus to reassure me. (P15)

However, many patients regretted a posteriori having received too little prior information about the severity of the possible side‐effects.

##### Faith

Although patients' faith in the procedure was based on the expertise of the doctors who had advised them, their trust in the success of the treatment was placed in the hands of God by those who were religious believers, whether they were Catholics, Protestants, Hindus or Muslims, ancestor worshipers or participants in spiritual rites of other kinds. They asked God directly to be cured, either in their prayers or via votive acts:

I handed over to God the Eternal, saying ‘If You want to take care of me, I have faith in You, it's up to You to cure me’. (P06)

##### Related donors

Having a family donor was perceived as an extra piece of luck because it would improve patients' chances of being cured. In addition, an intra‐family donation was held to be proof of “family solidarity”. However, one respondent would rather have had a non‐related donor so as not to feel indebted to the donor.

##### Peer testimonies

One patient decided to accept the allograft after hearing previously grafted patients' reassuring personal accounts of their experience:

The turning‐point (in the patient's decision‐making)… I think it was seeing the first‐hand accounts of people who had done it themselves which pushed me in that direction. (P22)

Some transplanted patients said they would like to describe their experience to patients who were hesitating. Bearing witness in favor of the graft was like making a return in exchange for the gift of life they had received.

##### Positive representations of the transplantation

Most of the respondents had understood the allograft procedure quite well and were able to describe it in their own words. The idea of ending up with new healthy bone marrow and blood was described as being equal to being given a new life:

It destroys all your own bone marrow. And the new substance takes over. (…). It's like a rebirth. Like when you were born, like a birth. (…). It's the same story, you are simply reborn. (P19)

The feeling of a “rebirth” took shape during the process and was rationalized by the patients after the transplantation. It therefore did not contribute to their decision, but it can nevertheless play an important role because grafted patients who bear witness can decisively influence those who are hesitating.

#### 3.2.3 Factors limiting patients' acceptance

##### Geographical distance

Overseas allografted patients have to undergo a long period of social isolation. In addition to the period of preventive isolation, which is part of the current transplantation process, overseas patients also have to stay on in mainland France after the graft, far from their families and friends. Four respondents said they had found it hard to make up their minds in fact because undergoing an allograft in mainland France meant leaving their families:

I remember having this discussion with my mother. I had said to her ‘No, I don't want to go’ because having the allograft meant going away to Paris for months and months. Being practically all alone (…). But later on, you see, I have two children (…) so I want to maximize my chances of living a longer life. If only for their sake. So I agreed in the end. (P15)

One patient reported, for example, that after having a relapse in 2018, she accepted the allograft only after some hesitation because of the distance involved. She admitted that if it happened again, she would not consent.

##### Apprehension of protective isolation

Several respondents declared that they dreaded the psychological effects of the protective isolation period. Those who had previously experienced a long period of isolation regarded this obligation as a further trial, which they would not want to undergo again if they were not sure of being cured:

Since I had just spent several months, well the days feel long in hospital, and I kept thinking: ‘more months ahead’. Then with time, one begins to think a lot harder. One says to oneself ‘what are …a few months out of a whole life? To be able to live on for ages, it's not such a huge price to pay, is it?’ (…) Yet one still feels these pangs of fear all the same: ‘I'm feeling fine just now. Why go through it once again? (P15)

##### Fear

Fear of dying if the transplantation was unsuccessful or in the event of lethal complications was another reason for not consenting, as stated by the daughter of the one non‐consenting patient:

We were speaking in terms of statistics, but he (the father) only grasped the fact that he might die (…): this hospital stay might end in his death if the graft was unsuccessful. (CR1)

Although this fear did not always result in a refusal, it caused some patients and their families to hesitate. One respondent also feared the psychological effects on the related donor if the graft failed.

##### Representations of chimerism

The graft was regarded by the one non‐consenting patient and by one caregiver (see Data S2) as an intrusive substance which threatened the recipient's identity:

I don't feel like putting things from other people into my body (…). I don't want them to be mixed up with mine. PR1

However, most of the respondents had a detached picture of chimerism, which has often been described as having partly transformed patients' personality. Some of them mentioned the physical and behavioral changes for which the chimeric process was presumably responsible:

I have his [his brother, the donor's] bone marrow, but since the transplant, things I didn't like before that he liked, well I'm starting to like them myself. Tattoos, for example. I would never have said to myself one day ‘I'm going to get a tattoo’. Whereas my brother was tattooed. The same goes with motorcycling. I didn't like motorbikes before. Now I'm on a motorbike practically every day. Whereas my brother likes motorbikes. (P15)

The latter extract does not reflect a negative representation of chimerism, but rather the feeling of making a new start in life.

## 4 DISCUSSION

The present findings show that the patients interviewed felt they had made a properly informed decision and that they had given their consent quite freely. Accepting the transplant treatment proposed was nevertheless regarded by many participants as an obvious choice since their decision was based not only on their own wishes but also on what was imposed on them.^21^ We have called this patient's feeling of having no choice “constrained choice”.

This choice was constrained in the first place by the feeling described by previous authors^1^, ^5^, ^6^ that allo‐HSCT was the only realistic therapeutic option available for improving patients' chances of long‐term survival. The logic of survival is particularly acute in the case of hematological cancer, the symptoms of which are strongly perceived and managed urgently at onco‐hematology departments. This logic of survival may outweigh the benefit/risk balance by attenuating patients' perception of the risks (of graft failure, serious complications, or relapsing) involved^22^, ^23^ and leading them to overestimate their chances of being cured.^7^, ^10^, ^11^ This way of interpreting medical information has been described as an optimistic bias and a form of adaptive behavior,^10^, ^11^ since the will to survive is inherent to the human condition.^5^, ^24^ In addition, the allografted patients interviewed here had consented to the transplantation and consciously accepted the risks, which they may therefore have voluntarily minimized.

Their choice was also partly constrained by pressures from family members urging patients to either accept the transplantation or not. Consenting to allo‐HSCT responded to the patients' need to go on playing their social role, especially their parental role. The strength of patients' commitment to their families is one of the most decisive factors on which their decision depends.^5^

This study confirms how important it is that physicians should provide their patients with medical information as completely and frankly as possible and build a trustful relationship with them, as previous authors have pointed out.^1^, ^3^, ^7^ The present findings show that patients' choice can be biased by the implicit benevolence of the physician. The respondents relied heavily on their physicians' advice when making their decision, as also reported in previous studies.^1^, ^3^ Since doctors are rarely completely non‐committal when making recommendations,^25^ they certainly carry considerable powers of persuasion. Patients' choices are therefore constrained by medical pressures, as well as by a kind of social desirability^11^ which makes them comply with the doctors' recommendations.

The present findings therefore corroborate those obtained by Forsyth et al.,^5^ since they confirm that obtaining patients' consent to allo‐HSCT is a complex process which is by no means restricted to the one‐to‐one exchange between a physician and his patient which takes place during the initial transplant evaluation visit. The decision‐making takes place in a relational setting including both the family circle and the medical team.

This process also involves individual socio‐cultural, experiential and geographical factors, which can either favor or detract from the wish to undergo the transplantation. Here we have highlighted some favorable factors which have received little attention in the allo‐HSCT literature, such as reliance on religious faith. Contrary to a common belief, turning to faith may not necessarily be synonymous with ineffective coping, passivity, and fatalism.^26^ Studies have shown that patients tend to trust their faith even more than the treatment to cure the disease.^27^ However, religious faith can also be a reason for not consenting to allo‐HSCT.^28^ Hearing about the personal experience of peers is another favorable factor. Patients feel better prepared for treatment and decision making when they have received experiential information.^29^ The possibility of receiving a family graft and patients' positive representations of the transplant process are other factors which can positively influence patients' decision making. Physicians are generally not very aware of these hitherto neglected factors because they concern patients' private spheres, which doctors do not generally take into account.

Fear of dying and fear of serious side‐effects are two other frequently combined factors which tend to make patients reluctant to consent. These factors rarely lead patients to refuse allo‐HSCT; however, because the benefit/risk ratio is biased by the hope of being cured.^7^ In addition, respondents felt with hindsight that they had not been sufficiently well informed in advance about the undesirable side‐effects of the transplantation. This does not necessarily mean that the information exchanged at the first medical consultation was incomplete: patients' selective memory may store only information sustaining their hopes because the emotional load associated with the consultation prevents them from remembering all the information delivered.^11^, ^30^ In addition, it was impossible for them to picture in advance what a painful experience they were about to undergo; being informed about side effects is not the same thing as experiencing them.^22^

Apprehension of social isolation and geographical distance are other limiting factors, which were specific to our sample of people inhabiting an island located a long way from the transplant center. These factors have rarely been addressed in the literature, except for a Canadian^12^ and an Australian^13^ study, although they are experienced like a two‐fold ordeal by many overseas patients.^17^ Geographical distance can also be expected to be a limiting factor in other situations, such as that of patients who need CART‐cell therapy or have to undergo a transplant for non‐malignant diseases such as sickle cell disease, or the problem of access to pediatric cancer care^31^ for children living far from a treatment center. Although consenting to allo‐HSCT was not actually enforced, patients from overseas territories and other very isolated regions felt that accepting the transplant treatment proposed was tantamount to submitting to enforced therapeutic mobility.^17^

### 4.1 Study limitations

The main limitation of this study is that our population included only one non‐consenting patient and the interpretation of our data was therefore restricted to this particular population. There are various possible reasons for this low non‐consent rate. First, it was due to the death of several non‐grafted patients. Second, some patients, amounting to a hitherto undetermined percentage, refused the transplantation before being referred to the transplant hematologists. A previous US study showed that having received the first course of treatment at a non‐academic hospital was associated with a lower rate of HSCT.^32^ In order to include all the patients who needed an allo‐HSCT (those who would accept as well as those who would refuse), the patients in this study could have been interviewed after the initial transplant evaluation visit. However, this change in the study design might have an ethical impact because the interview guide used to explore the reasons for the patients' choice might have affected the decisions they made.

Another limitation of this study is that all the patients were interviewed after the transplant and not during the graft process. Most of the patients (14/22) were interviewed more than a year after the transplant (see Table 1). Their narratives were therefore reconstructions of events, but they still reflected their lived experience. In order to restrict the possible memory biases in patients' initial experience of decision‐making, the interviews could have been conducted nearer the time of the transplant, but this would have considerably reduced the number of participants included in this study.

### 4.2 Clinical implications

The topic of patients' consent to allo‐HSCT should not be addressed simply in terms of the communication between physicians and their patients and the need to improve patients' understanding of the allograft and the benefits and risks involved. Other factors favoring or limiting patients' acceptance, such as their personal values and representations, should be taken into account in a shared decision‐making perspective. The physician should help patients and their families to explain their values and preferences more clearly, as well as their perceptions and representations of the disease and the treatment proposed in order to adapt the information exchanged accordingly.^2^

On the other hand, since social isolation and geographical considerations can influence patients' decision, several efforts need to be made to relieve this isolation and improve the means of providing family support, by introducing digital devices, for example. The latest telepresence robots, for example, are highly promising long‐distance tools for sustaining social relationships and relieving the psychological burden of isolation.^33^, ^34^ Their beneficial effects on the continuation of parenthood are currently being studied.^35^

## 5 CONCLUSION

The present study can help to understand the factors affecting patients' decision to accept allo‐HSCT. Among the factors identified in this study (the medical information received, faith, having a family donor, peer testimonies, representations of the transplantation and chimerism, remoteness, apprehension of protective isolation, fear of dying), which sampled people living on an isolated island, the geographical distance between patients' home and the transplant center is one of the main issues underlying the acceptability of innovative or highly technical treatment for many patients living in French overseas regions, as well as other remote areas worldwide, who have to be relocated to a highly specialized hospital located far away.

Further studies are now required to elucidate the reasons why allo‐HSCT was refused by some patients, especially those who refused before the initial transplant evaluation visit.

## AUTHOR CONTRIBUTIONS

**Aline Sarradon‐Eck:** Conceptualization (equal); formal analysis (equal); methodology (equal); supervision (equal); writing – original draft (equal). **Loreley Franchina:** Data curation (equal); formal analysis (equal); investigation (equal); methodology (equal); writing – original draft (equal). **Yolande Arnault:** Writing – review and editing (equal). **Anne‐Gaëlle Le Corroller:** Writing – review and editing (equal). **Patricia Zunic:** Resources (equal); writing – review and editing (equal). **Patricia Marino:** Funding acquisition (equal); project administration (equal); validation (equal); writing – review and editing (equal).

## FUNDING INFORMATION

This qualitative study was funded by the Institut National du Cancer (National Cancer Institute)‐INCa “Projet libre de recherche en sciences humaines et sociales, épidémiologie et santé publique” 2019 (SHSESP‐19‐174).

## CONFLICT OF INTEREST STATEMENT

The authors have no conflicts of interest to declare.

## ETHICS STATEMENT

This study was approved by the French National Institute for Health and Medical Research Institutional Review Board (IRB00003888, No. 19‐631). Patients and family caregivers were given oral and written information before taking part in the study.

## Supporting information


Data S1.

Data S2.

## Data Availability

The data that supports the findings of this study are available in the supplementary material of this article.
