# Supplementary material for: Consenting rather than choosing. A qualitative study on overseas patients' decision to undergo hematopoietic stem cell transplantation
Source: Cancer Med. 2024 Jan 9;13(1):e6934. doi: 10.1002/cam4.6934 (PMC10807688; doi:10.1002/cam4.6934)
Supplement: Supplementary file 1 — Data S1. [file CAM4-13-e6934-s002.docx]

**Interview Guides**

General comment: these were semi-directive interviews, which means that they were based on the following list of themes relating to the study. The aim was to help respondents express themselves by suggesting these themes, while still leaving them some freedom of expression. Questions in brackets are suggestions which could be used to prompt respondents if need be. The order in which topics were addressed could vary, depending on how the interview was going. The questions were not necessarily all covered, as they were simply intended to guide the interviewers. Although the themes addressed were mostly chosen in advance, additional topics could be added from one interview to another until data saturation was reached. The respondents were encouraged to adopt an easy narrative tone when talking about their own personal experience.

**Patients who had undergone an allo-HSCT**

*Patients’ knowledge and understanding of the disease and the various treatments available*

- Can you please explain in your own words how your illness started and how it affected your body?
- What treatments did you undergo before having an allograft?
- Can you tell me in your own words how these treatments worked?
- Can you tell me in your own words how an allograft works?
- Why do you think an allograft was proposed to you?
- At what stage in your treatment was the idea of an allograft first mentioned?
- Who was the donor?

(How was the donor chosen? Was the donor a relative of yours)?

*Psychosocial aspects of the transplant*

- Can you tell me what the graft procedure involved?

(When did it occur? When did you leave La Réunion?) What did the operation involve? What were the side-effects? What was the hardest part for you? How did you experience your discharge from hospital? Where did you stay after leaving the hospital? How satisfied were you with the care you were given at that stage? What were the financial effects of having to travel to mainland France and undergoing an allograft?)

- What improvements could be made, in your opinion?

*Psychosocial experience of the long period of separation from the Island, one’s family, one’s work and going back home*

- How long were you separated from your friends and family?
- How did you experience this separation?
- What did you miss most?
- Can you tell me how you experienced going back to live at home?
- How did you experience the care you received after leaving mainland France? How satisfied were you with this care?
- What improvements could be made, in your opinion?
- [if the patient was in activity before contracting the disease] Have you gone back to work? Under what conditions? How did you experience your return to work?

*Information received by patients about allo-HSCT*

- Do you feel you were given all the information you needed (at the Reunion University Hospital? And then at the transplant center? Was enough time devoted to providing you with this information during consultations?)
- Did you understand all the information you received?
- Did you seek further information (via Internet, patients’ associations, books, etc.)?
- What improvements could be made, in your opinion?

*Factors involved in patients’ decision to undergo an allo-HSCT*

- Did you hesitate before deciding whether or not to undergo an allograft? (whatever your final decision was): for what reasons?
- What was the most decisive factor involved in making your decision? (Feeling free to decide? Discussions with the medical team? Sharing the decision-making with others?)
- Did you ask your friends and family for their opinion?
- Before making your decision, did you meet other people from La Réunion who had undergone the graft?
- If the graft had been available in La Réunion, would that have affected your decision?

*Patients’ sociodemographic data*

- Age, educational level, occupation, marital status, number of children
- Previous stays in mainland France
- Belonging to any particular religious or cultural community
- Did you have any close friends or relatives living in mainland France who might have helped?

**Carers of the patients who had undergone an allo-HSCT**

*General and sociodemographic particularities*

- What is your relationship with the patient?
- Have you any brothers and sisters?
- What other people in the patient’s family circle have acted as carers?
- When did you start helping or accompanying the patient?
- Do you have an occupational activity?
- Age, educational level, occupation, marital status, number of children
- Previous stays in mainland France
- Are you a member of any particular religious or cultural community?
- Were you yourself the donor (if not, who was the donor?)

*Carers’ psychosocial experience of the graft*

- Did you travel with the patient to mainland France? How long did you stay there?
- Can you tell me what the patient’s graft procedure involved? (When was it performed? When did the patient leave la Réunion? What did the patient’s care consist of? In your opinion, what were the most painful or unpleasant effects of the graft on the patient? How did the patient’s discharge from hospital go? Where did the patient stay after leaving hospital? Do you think the care received by the patient at that point was satisfactory? What were the financial consequences for the patient of having to travel to mainland France and undergoing the graft?)
- What improvements could be made, in your opinion?

*Patients’ psychosocial experience of the long separation from their family and their occupational environment and their return to their family circle*

- How long was the patient separated from his/her friends and family?

How did he/she experience this separation?

- Can you tell me what happened when the patient went back to live at home?
  What improvements could be made, in your opinion?

*Carers’ experience of helping and accompanying an allo-HSCT patient*

- What role did you play during the patient’s illness?

(providing logistic, domestic, financial and/or psychological support, medical care, etc.)

- What do you think was the most important part of the assistance you provided? (moral support, logistic support, financial assistance, etc.?)
- Did this assistance have an impact on your own everyday life? (in what ways?)
- Did this assistance affect your working life? (taking leave from work, having no holidays, relationships with colleagues, working hours?)
- Did it have an impact on your social life? (relationships with friends and family, social and leisure activities, projects?)
- Did it have an impact on your health (your well-being, mood, sleep, appetite, tiredness, medical attention)?

*Carers’ knowledge and understanding of the illness and its treatment*

- Can you please explain in your own words what you have understood about this illness?
- What treatments did the patient undergo before having the allograft?
- Can you tell me in your own words how these treatments work?
- Can you tell me in your own words how an allograft works?

*The information received about allo-HSCT*

- Do you feel you and/or the patient were given all the information you needed (at the Reunion University Hospital? And then at the transplant center?)
- Did you understand all the information you were given?
- Did you seek further information (via Internet, patients’ associations books, etc.)?
- What improvements could be made, in your opinion?

*Factors involved in the patient’s decision to undergo an allo-HSCT*

- Did he/she hesitate before deciding whether or not to have an allograft (whatever their final decision was)? For what reasons?
- What was the most decisive factor involved in the patient’s decision?
- Did he/she ask for your opinion? Did you give your opinion (whether you were for or against the allograft)?

Would he/she have hesitated if the graft procedure had been available at a hospital in La Réunion?)

**Questions to patients who refused to undergo an allo-HSCT**

*Patients’ knowledge and understanding of the illness and its treatment*

- Can you please tell me in your own words how the disease started? How did it affect your body?
- What treatments have you undergone?
- Can you tell me in your own words how these treatments work?
- How satisfied were you with the care you were given?
- Can you tell me what the hardest part of this care was for you?

*Information received about the allo-HSCT procedure*

- At what stage was the possibility of having an allograft mentioned?
- Do you feel you were given all the information you needed about the allograft?
- Did you understand all the information you received?
- Did you seek further information? (via Internet, patients’ associations, books, etc.)?

*Factors involved in the patient’s decision to refuse the allo-HSCT*

- Did you hesitate when the idea of having an allograft was first suggested (whatever your final decision was)? For what reasons?
- When did you refuse this proposal and why? (before or after the research of a potential donors?)
- What were the main factors on which your final decision was based?
- Did you ask your friends and family for their opinion?
- Have you met any other people from La Réunion who have undergone an allograft?
- Would you have agreed to having an allograft if the procedure had been available at a hospital in La Réunion?

S*ociodemographic data of patients who refused the allograft?*

- Age, educational level, occupation, marital status, number of children
- Previous stays in mainland France
- Are you a member of any particular religious or cultural community?
- Did you have any close friends or relatives living in mainland France who might have helped?
