# Supplementary material for: Consenting rather than choosing. A qualitative study on overseas patients' decision to undergo hematopoietic stem cell transplantation
Source: Cancer Med. 2024 Jan 9;13(1):e6934. doi: 10.1002/cam4.6934 (PMC10807688; doi:10.1002/cam4.6934)
Supplement: Supplementary file 2 — Data S2. [file CAM4-13-e6934-s001.docx]

**Part of the codebook focusing on decision-making**

| **Themes** | **Sub-themes** | **Examples of typical statements** |
| --- | --- | --- |
| A  constrained choice | The feeling of being in a therapeutic impasse | She had little choice anyway, because (…) the only thing that could really cure her was having the graft. It was either the graft or nothing. C05  Well naturally, they let me decide, but I felt as if I had no choice. I had to do it to prevent the risk of relapsing. It felt like part of the whole procedure, so it just had to be done. P12  There was no other alternative. He (physician) said ‘the only way of being sure of surviving is to go for the graft. You don’t have hundreds of other options’. C09  Well in any case, I didn’t have much choice, did I? The only choice open to me was either to go on taking Vidaza^®^ and I would only have a year left to live. Or else to have the allograft. P20  It turned out that the disease had already progressed considerably. I was in the final stage after all. That was when they started to apply the graft. I didn’t even want to be treated any more. But the doctor convinced me by saying that I was still young, that I had my children and my grandchildren to think about, so I had to carry on. So, I did start to think and I said all right then. P03  You could see the disease was progressing after all. So then she was told ‘you will have to make the journey’, there’s a fifty-fifty chance of surviving, in any case. C03b |
|  | A survival strategy | I had no hesitation because it was either staying put here (in La Reunion) and going on with the chemotherapy and that would mean…certain death. Or making the journey over there (to mainland France) and taking my chances. You get either life or death, so I decided… I took the best decision in the hope of surviving. P05  (The doctor) suggested. (…) If I don’t agree, I am going to die. But if I do agree, it has to be done, (…) I have no other choice. I have no choice. It has to be done. Yes, it has to be done. It’s a matter of life, a matter of life. If you want to survive, you have to do it. P17  When you know you are going to die otherwise, what risk are you taking? No risk at all. I always knew there would be a few little problems (complications). Not such big ones, though. But I’m still here, I’m still alive. And that’s what matters. P19  I realized that I was obliged to do it. I wasn’t really obliged to do it, but if I wanted to stay alive, it was really the best thing to do (…). I wasn’t sure it would work, but at least I will have tried. P21 |
|  | Surviving for their family’s sake and family pressures | I have two children, and I said to myself: ‘I wouldn’t want…I mean, I want to maximize my chances of living as long as possible. If only for them’. So then I agreed to go ahead. P15  I want to go on living. My fight is for living. Living not just for myself, but for him (her little boy) because I must help him to live. Because I want to pass on everything I know to him. P18  My son said to me ‘Mummy, you must get yourself treated. I still need you’. P03  My father didn’t want me to have it (allo-HSCT) but my mother wanted me to have it. It’s a decision I took all on my own, a well thought-out decision. P22 |
|  | Doctors’ pressures | It was I who decided, but he (the doctor) advised me quite forcibly. (...) He presented it as the only possible option, although he said I could make up my own mind, but in the end, he made me understand on the contrary that I didn’t really have any choice. P22  Patients only rarely refuse to have the graft. If they do refuse, (…) it’s because we ourselves are not quite at ease with what we propose and they are able to sense it (…) The doctor has a highly convincing role to play, I can see that! Especially when there are diseases of that kind which are liable to be lethal (…) If one thinks when patients refuse that they are losing a great opportunity, we will push the point in that case, but if we are faced with a refusal and we don’t feel too happy about taking that path, (…) we are not going to insist, are we? (...) We try to give them all the keys to help them choose, well not exactly choose but make a decision. N6 |
|  | Being managed | There’s an expression we have here, which goes ‘when you are in the dance, you dance’, so there was really no choice… There was the graft available, all the expenses were covered as well, so the only thing to do was to go ahead with it. And we will see what the outcome is afterwards. P07  To my mind, it was part of the whole procedure, one just had to go along with it. At one point, I wondered whether I should really do it or not. He (the physician) said to me ‘Of course you must do it, that’s how it is and there’s no other way about it’. It was part of the whole procedure. There was no shilly-shallying. P12 |
| The semantic register of the decision-making | Being active in the process | It’s something you have to decide for yourself. I was given the possibility of having a bone marrow graft and I again contracted a moral agreement with the doctors. (…) One shouldn’t just undergo what is happening to us. One must be a real decision-maker. P04 |
|  | Trust | I took the decision to live when talking to my doctors. I said ‘I trust you two hundred percent’. (…) My aim was to reach the final goal (…) I came to an agreement, psychologically speaking, with the doctors, in a contract about a long-term healing process. P04 |
|  | Luck | When one is given a chance of being restored to life, one must go for it. That’s it. P03  There was one (100% compatible donor), so what can you do? A chance in a million, you cannot let it go obviously. C09 |
|  | Fight | No. No. No, no (without any hesitation). I went for it quite wholeheartedly. But then, I am a warrior at heart! P19  I said to myself: ‘It’s a trial, just another trial, and we are going to win it. Now we are going to war! We have won three battles and now we are going to win the war’. C11 |
| The factors favoring patients’ acceptance | The medical information received | We were well supported in terms of the doctors’ advice, about how everything was going to be done. (…) So I would say no, I had no doubts about that particular treatment. C14  I had a doctor who explained the graft extraordinarily well to me and gave me confidence in going ahead. P09  He (the doctor) asked me to arrange meeting between him and my family (…). To explain the positive and negative aspects possibly involved in having the graft. (…) He took the time to explain to the family what was going to happen and the consequences. P05  To all the questions I asked they gave me the answers. And even before asking these questions, I already had the answers, and that was…really important. (…) Thanks to these discussions, I never had the feeling that I was going to die (…) The idea that it might not work just never entered my mind. I am sure it was thanks to the doctors. They managed to advise and thus to reassure me. P15  We took the decision – well I mean… we chose the option in favor of the graft. And we discussed everything with the doctor in order to look at all the aspects. In order to definitely confirm the decision. I must admit that I was extremely…how can I say it? Perplexed, actually, or even undecided at this stage (…). Because at the time when we took the decision, I was feeling I had recovered all my strength. I was in good form, I was going out running, I was feeling great. Yes but (that was thanks to) the discussions with the team of doctors (…) who were there to support me (…). But when I asked: ‘but if we decide today to opt for the graft, can you assure me that I will actually be cured 100 per cent?’. He was very honest with me, and said ‘no, that would be lying to you (laughter). It would be a lie to say you will be cured 100 per cent. I can only say yes and no. Yes, it’s possible; it will depend in the end on your physical condition, your body, and your mental state especially’. This honesty, this frankness on the part of the doctors in charge of me who told me that there was never a one hundred percent positive response, that there was no 100% likelihood of my being cured which made me decide in the end because they were telling me the truth in a way. They were not hiding the truth from me during our discussions’. P01 |
|  | Faith | So I said ‘Now I am going to go through with the whole procedure’, which is what I did. And since I am a religious person, I prayed ‘Lord, give me the strength and courage to overcome this disease’ and I decided to go through with the whole procedure. P08  But faith is a great thing. One must hang on there. You mustn’t give up, you must forge ahead. Take a firm hold of your rosary and there you are. P19.  I handed over to God the Eternal, saying ‘If You want to take care of me, I have faith in You, it’s up to You to cure me’. P06  I had some rather bad luck, let’s say (…): before the operation (the graft), we went through, I mean, we had some bad times. So we said: there is something wrong here. So we got into closer touch with that religion (the Reunionese Hindu one) before setting off (for mainland France to have the graft). P07 |
|  | Having a related donor | I had a brother who was 50% compatible with me. Well 50% may not seem much, but having a 50% compatible brother is great. (…) I said ‘There you are, now you know the situation? What would you do? If you were in my place, what so you think you would do?’. They (the doctors) answered ‘We would go ahead with it’. It was perfectly clear. ‘We would go ahead with it because your brother is there and he is compatible just now. Tomorrow you never know’. (…) The proof that they were right is that one year and a half later, my brother had an attack (…). So we did it at the right moment after all. P04 |
|  | Peer testimonies | The turning-point (in the decision-making), I think it was seeing the first-hand accounts of people who had done it themselves which pushed me into that direction. P22  I have heard there are people who don’t want to go and be treated, which I think is such a pity. I would like to go and meet these people and find out why. If they are afraid, for example, of traveling to mainland France, I would say no, you must go (…), and perhaps nobody will ever refuse again to go, at least I hope not. And I said to the doctor that if there are cases like that, if they want me to come and talk to them, it would be no problem for me, on the contrary. I was on the receiving end myself, but now I can give hope to people who have a patient in their family (…); I have seen that bearing witness can have an impact. P10 |
|  | Positive representations of the graft  Chimeras  Acquiring new bone marrow  A rebirth  The gift of life | We are chimeras, as the etymology of the original Greek word ‘chimera’ shows. It’s about two different organisms living together (…), which cohabit but not always peacefully. Even we humans are not capable of cohabiting properly with our neighbours without coming to blows (laughter). Likewise in the same body, it’s not always easy (…). It’s not easy to wake up in the morning thinking ‘damn it, my blood group has changed, and I no longer have the same DNA as before’. And then there is somebody else living inside me’. P04  Roughly speaking, it’s a question of (…) replacing my bone marrow with healthy bone marrow, new bone marrow. And that new bone marrow is going to produce cells for me and release them into my bloodstream. P21  The reason for the allograft is to destroy my own bone marrow, which is deficient and unhealthy. And the purpose of the new bone marrow is to take over the job… to take over inside my body, giving me a new life, one might say (laughter). Yes, having a brand new healthy marrow devoid of leukemia. P05  It destroys all your own bone marrow. And the new substance takes over. (…). It’s like a rebirth. Like when you were born, like a birth. (…).. It’s the same story, you are simply reborn. P19  I gave birth myself 20 years ago, and now it’s that pocket which is going to produce a new life. It’s a rebirth, the rebirth of Adèle. C11  In a way, one is reborn. Now I have two birthdays…the date of my graft is like a second birthday for me because without the day of that graft, we would no longer be where we are today, and my son would maybe be motherless by now. P10  I’ve got my brother’s bone marrow, and that is what has kept me alive. It’s thanks to him that I am still here. P14  It’s like a second life. That donor has given me a second life. I pray for that donor every day. I pray for him because (…) without him, who knows what was going to become of me? I would be dead by now, perhaps. And during this whole new life until I die, I am going to pray for that person (with tears in her voice). P16 |
| Factors limiting patents’ acceptance | Geographical distance | In my hospital room, the person next to me refused to have the bone marrow graft. Why do people refuse? There can be several reasons. First of all, they are Reunionese. In this part of the world, if people are told ‘you must go, off you go to mainland France’, their immediate reaction is ‘oh no!’ It’s not because of the bone graft, though. It’s for any old reason. It’s because these people were born like that on these islands (…), it’s hard for them to leave their families. Some of them even refuse to go because of that. P04  I remember having this discussion with my mother. I had said to her ‘No, I don’t want to go’ because having the allograft meant going away to Paris for months and months. Being practically all alone (…). But later on, you see, I have two children (…) so I want to maximize my chances of living a longer life. If only for their sake. So I agreed in the end’. P15  The only thing he focused on about the possibility of having this graft was the need to travel to mainland France. CR01 |
|  | Apprehension of protective isolation | Since I had just spent several months, well the days feel long in hospital, and I kept thinking: ‘more months ahead’. Then with time, one begins to think a lot harder. One says to oneself ‘what are …a few months out of a whole life? To be able to live on for ages, it’s not such a huge price to pay, is it?’. Especially as it was for my own good’. Yet one still feels these pangs of fear all the same: ‘I’m feeling fine just now. Why go through it once again?’. P15 |
|  | Fear | We were speaking in terms of statistics, but he (the patient) only grasped the fact that he might die (…): this hospital stay might end in his death if the graft was unsuccessful. CR01  At the beginning, she wasn’t too keen on having the graft because in fact, the idea of a graft meant to her that she was going to die. CA03b  She was highly motivated at first (…). Then later on, we were given a little booklet. And part of this booklet was about the graft, its side effects, and there were some first-hand accounts. The risks were presented in full detail. (…) So we were all highly motivated, (…) until she read the booklet. And suddenly, I had never seen her like that, she was full of doubts. (…) She didn’t know whether to go on with it. Whether to take the risk. At first there was even a period of denial, too. Had she really had that relapse or not (…)? Perhaps the chemotherapy will work now and the disease will never return. C21  They say the graft is a way of being cured, but there are lots of risks involved, is it worth taking all those risks? (…) I have been having doubts because in fact (…), I’m scared. They tell you ‘We’re going to perform a graft on you, but we are not sure whether it will work’. P09  Then (…) when the donor is one of the family and the graft doesn’t work, the donor often feels he or she is to blame because it hasn’t worked. And I have been wondering whether my little sister (the donor) would be able to cope with that. P21 |
|  | Negative representations of chimerism | I don’t feel like putting things from other people into my body (…). I don’t want them to be mixed up with mine. PR01  I was against having the graft. Really completely against it. It made me feel hyper-scared. Scared that it might change his body, that it might no longer be him. (…) Human bodies are all unique. So although they talk about ‘100% compatible’, we are not made to receive other people’s cells. That’s why those tons of medicine have to be prescribed! So I have been thinking ‘it’s going to be an internal battle. It’s going to be horrible’ (…). All his life, he is going to be fragile, yes, fragile. C09 |
